# Emotional vigilance under perceived continuous evaluation in foreign language classrooms: evidence from Chinese university students

**DOI:** 10.3389/fpsyg.2026.1793299

**Published:** 2026-05-12

**Authors:** Zhihui Ding, Guoxing Bu, Quan Fang

**Affiliations:** 1 Shanghai University of Medicine & Health Sciences, Department of Foreign Languages, Shanghai, China; 2 Dongshin University, Naju-si, Republic of Korea; 3 Xinjiang Normal University, Ürümqi, China

**Keywords:** context exposure, evaluative classroom, perceived learning outcomes, primary emotional vigilance, secondary emotional vigilance

## Abstract

Evaluation activities in foreign language classrooms have increasingly become embedded in everyday instructional practice rather than remaining occasional measurement events. Yet empirical evidence remains limited regarding how students' perceived exposure to continuously evaluative classroom conditions is associated with learning-related functioning through emotion-regulation processes. Drawing on data from Chinese university students in foreign language learning contexts, this study examines an integrated model linking contextual exposure, primary emotional vigilance, secondary emotional vigilance, and perceived learning outcomes. The results show that contextual exposure is positively associated with perceived learning outcomes across the sample. Primary emotional vigilance emerged as the main mediating mechanism through which perceived evaluative classroom conditions were associated with learning-related functioning, whereas secondary emotional vigilance showed an additional positive contribution in the full model. Multi-group analyses further indicate that these associations remain broadly consistent across learner subgroups, suggesting that evaluative classroom conditions may operate as a shared organizational feature rather than a pattern driven primarily by group-specific differences. Overall, the findings suggest that learning in evaluative foreign language classrooms is associated not only with linguistic development but also with students' psychological adaptation to conditions of visibility, feedback, and judgment. These results highlight the importance of assessment designs that preserve instructional effectiveness while also supporting psychologically sustainable classroom evaluation practices.

## 1 Introduction

### 1.1 Continuous evaluation in global education: how intensified assessment becomes an everyday classroom context in foreign language learning

In many education systems, assessment has moved from periodic examinations to practices that accompany learning throughout the instructional process. Rather than being limited to formal testing occasions, evaluation now appears routinely in everyday classroom interaction. In second and foreign language education, learning-oriented assessment has gained wide acceptance as an approach to improving feedback quality and learner engagement. At the same time, it has increased the density of evaluative cues in daily classroom life, placing learners in settings where performance is continuously open to observation, comparison, and feedback (Turner and Purpura, 2016). This development has been reinforced by policy agendas that prioritize measurable learning outcomes and institutional accountability. In the Asia–Pacific region, language education policies have placed growing emphasis on standardization, outcome-based instruction, and performance evaluation, leading to foreign-language classrooms in which continuous assessment structures daily teaching practice (Majhanovich, 2014). Comparative research on English education in East Asia further indicates that global educational discourses, once adapted to local contexts, often take shape as classroom arrangements marked by competition, ranking, and sustained evaluation (Choi, 2016). In classroom practice, technological and pedagogical innovations have further heightened the visibility of assessment. Online learning platforms, video-mediated instruction, and instant feedback tools allow teachers to record, track, and respond to learner performance in real time. Evaluation therefore operates increasingly as an ongoing interactive process rather than a discrete instructional event (Whyte, 2011). At the same time, constraints in implementing learning-oriented assessment frequently push teachers toward intensive monitoring and frequent feedback, leading learners to experience evaluation as a persistent classroom presence rather than solely as supportive guidance (Jalilzadeh and Coombe, 2023). Even in heritage and foreign-language programs framed around developmental aims, assessment retains strong evaluative functions that shape how learners approach participation and performance expectations (Son, 2017). Continuous assessment has accordingly become an internal organizing feature of foreign-language learning environments rather than an auxiliary classroom component. This classroom condition motivates an empirical examination of how students' perceived exposure to evaluative settings is associated with emotional vigilance and learning-related functioning.

### 1.2 From foreign language anxiety to emotional vigilance: theoretical trajectories of evaluation, self-monitoring, and fear of negative judgment

Research on emotional processes in foreign language learning first emerged from investigations of foreign language classroom anxiety. Early studies highlighted how fear of negative evaluation, performance pressure, and doubts about linguistic competence interfere with learning performance and classroom participation. Interaction-based classroom research has further shown that when learning activities are closely linked to real-time feedback systems, evaluative signals become embedded in teacher–student exchanges, placing learners in situations where speaking and responding are continuously open to judgment (Chang and Lin, 2019). Intervention research addressing this condition suggests that although anxiety-reduction programs can lower visible tension in language classrooms, evaluative contexts continue to maintain an underlying state of psychological alertness rather than eliminating emotional demands from the learning process (Toyama and Yamazaki, 2019). As attention turned to more specific psychological mechanisms, longitudinal evidence began to show that the link between classroom anxiety and language achievement changes over time. Cycles of self-monitoring and anticipation of negative evaluation gradually reinforce this association (Liu, 2021). During the shift to online language learning in the pandemic period, digital teaching environments further increased the visibility and traceability of learner performance. Cameras, platform records, and instant feedback interfaces encouraged enduring patterns of self-scrutiny and expectation of evaluation (Yuan, 2023). Later work extended inquiry beyond anxiety to achievement-emotion perspectives, demonstrating that mindset and academic engagement are shaped by emotional processes triggered by perceived evaluation, involving not only tension but also sustained self-attention and emotion regulation (Ebn-Abbasi et al., 2024). Complementary phenomenological studies of teachers likewise indicate that in evaluation-intensive classrooms, emotional labor becomes a routine means of sustaining interaction and order, suggesting that emotional management has become part of the internal functioning of classroom life (Jalilzadeh et al., 2024). Taken together, these lines of research map a shift in focus from anxiety, to achievement-related emotions, and to emotional labor in language classrooms. Current explanations, however, concentrate primarily on emotional intensity or regulatory strategies. They leave open the question of why learners exposed to continuous evaluation develop enduring sensitivity to evaluative cues and stable self-monitoring tendencies. In this context, the concept of emotional vigilance offers a way to describe the psychological organization of learning under sustained evaluation and addresses a theoretical issue that has emerged from this body of research.

### 1.3 Reconsidering emotional vigilance in evaluative classrooms: unresolved links between context exposure and learning functioning

Recent work in foreign language learning has increasingly examined how motivation, emotion, and learning outcomes interact rather than treating emotional variables in isolation. Empirical studies consistently report that motivation, self-efficacy, and positive emotional experiences are closely associated with learner engagement and achievement. For instance, research in online language learning shows that grit and enjoyment shape learning investment through self-efficacy (Derakhshan and Fathi, 2023). Studies of mobile language learning likewise indicate that combinations of learning strategies and motivational orientations contribute to differences in learning performance (Yu et al., 2022). Related findings among multilingual learners further demonstrate that self-efficacy beliefs and strategy use are linked to academic attainment (Calafato, 2023). Alongside these developments, instructional research has begun to explore how emotional features of learning environments influence classroom experience. Work on digital and online instruction has shown that emotional affordances embedded in learning design affect learners' engagement patterns (Park and Lim, 2018), while AI-mediated feedback systems have been found to shape emotional stability and writing development in technology-supported classrooms (Mohammed and Khalid, 2025). Broader motivational studies also suggest that global competence and language learning motivation are increasingly situated within competitive and evaluative social settings (Semaan and Yamazaki, 2015).

Despite these advances, existing explanations tend to focus on emotional intensity, emotional categories, or regulation strategies. Less attention has been given to how learners exposed to continual evaluative cues in classroom contexts develop enduring self-monitoring tendencies and sustained expectations of evaluation. The ways in which contextual exposure and emotional processes operate together within a single explanatory structure therefore remain insufficiently specified. Building on this issue, the present study introduces emotional vigilance and proposes an integrated account linking contextual exposure, differentiated forms of emotional vigilance, and learning-related functioning. It examines how primary and secondary emotional vigilance are associated with learning-related functioning under perceived continuously evaluative classroom conditions.

## 2 Review

### 2.1 Evaluative classroom contexts as structured exposure: from assessment practices to perceived context exposure

Foreign language classrooms have often been examined as instructional spaces shaped by particular patterns of discourse and interaction. Turn-taking routines, feedback exchanges, and assessment practices work together to influence how learners experience classroom participation. Reviews of classroom discourse research in language education show that interaction is structured through teacher-initiated sequences of questioning, responding, and evaluating rather than through neutral conversational exchange. Such interactional arrangements place learners in situations where their contributions are regularly open to observation and assessment (Thoms, 2012). Early investigations of text-oriented language classrooms likewise showed that teacher-controlled discourse flow and evaluative feedback form the basic structure of classroom participation, situating learners' language production within repeated cycles of correction and appraisal (Mantero, 2002). In many Asian educational settings, close analyses of teacher–student dialogue indicate that IRF interaction patterns sustain the prominence of teacher evaluation in classroom activity. After each learner contribution, classroom talk typically moves into sequences of appraisal and confirmation, encouraging ongoing awareness of evaluative expectations (Liu, 2008). Later studies report that even when classrooms incorporate first-language support or more flexible discourse strategies, IRF structures continue to guide participation, leading learners to encounter similar exposure to assessment across different language-use contexts (Li, 2018). Research on collaborative learning designs further suggests that, even in settings emphasizing peer interaction and cooperation, teacher evaluation and classroom norms remain influential in shaping learners' perceptions of participation risk and performance security (Sun and Yuan, 2018). Critical analyses of learner-centered pedagogy have also shown that shifts in instructional ideology do not eliminate classroom role asymmetries or power relations. Responsibility for evaluation continues to rest largely within teacher-led discourse practices, and learners often experience implicit expectations of assessment even in interactional environments framed as open and participatory (Le Ha, 2014). Evaluation practices in foreign language classrooms therefore function as more than procedural features of instruction. They shape how learners interpret classroom situations, making contextual exposure a useful concept for understanding experience in evaluative classroom environments.

### 2.2 Emotional vigilance under continuous evaluation: mechanisms of self-monitoring and anticipated judgment

Research on social and emotional learning has consistently examined how learners manage emotions, interpret social signals, and maintain engagement in educational settings. Classroom emotional experience is not simply an individual reaction but develops through interactional arrangements and shared expectations. Studies of learner groups indicate that classroom environments generate different emotional demands and patterns of regulation. Under performance-related conditions, learners become increasingly attentive to social cues linked to evaluation (Smith, 2017). Work on teachers' emotional competence further shows that emotion regulation and classroom interaction quality shape learners' sense of psychological safety and awareness of evaluation, influencing tendencies toward self-monitoring during participation (Collie, 2017). Conceptual frameworks of social and emotional learning describe how learners gradually acquire ways of interpreting feedback, adjusting self-expression, and anticipating social judgment. Through these ongoing processes, emotional management becomes part of the psychological organization of classroom activity (Collie et al., 2017). Studies conducted in Chinese educational settings report that social and emotional learning practices reinforce sensitivity to social norms and expectations, making emotional regulation and self-restraint regular features of classroom participation (Yu and Jiang, 2017). Research in Korean classrooms similarly indicates that emotional learning programs help students handle academic pressure while increasing attentiveness to evaluative situations, leading to continuing self-monitoring and behavioral adjustment during performance tasks (Lee and Bong, 2017). Integrative reviews further suggest that social and emotional learning is reshaping emotional and relational dynamics in classrooms, as learners in collective learning settings learn to anticipate others' reactions and manage emotional presentation (Martin et al., 2017). Overall, this body of research suggests that classroom emotional processes involve more than emotion regulation strategies alone and extend to sustained awareness of social evaluative signals and self-regulatory practice. In classrooms where evaluation is continuous, these conditions heighten learners' sensitivity to judgment cues, making emotional vigilance a useful concept for understanding psychological functioning in evaluative learning environments.

### 2.3 Dual-process emotional pathways in learning: distinct functions of primary and secondary emotional responses

Research in learning psychology increasingly shows that learning outcomes emerge from the interaction of multiple psychological processes rather than from any single emotional factor or learning strategy. Early investigations of learning styles and learning strategies demonstrated that learners adopt different psychological response patterns during task engagement, information processing, and performance regulation, and that these patterns influence achievement and task completion quality (Feng et al., 2019). Studies of educational organization have likewise shown that professional learning communities function as support structures for teachers and learners, where variations in support practices shape experiences of emotion regulation and the maintenance of self-efficacy (Pang and Wang, 2016). The introduction of flipped and blended instruction into foreign language education has further highlighted how classroom restructuring alters learners' emotional investment before, during, and after class. Changes in instructional design activate different psychological response modes across learning stages (Fisher et al., 2024). Evidence from flipped language classrooms indicates that learners display distinct forms of emotion regulation and participation motivation in autonomous learning phases and in classroom interaction, suggesting parallel psychological operations within the same learning process (Alhamami and Khan, 2019). Research on technology-supported language learning also shows that corpus-assisted reading and vocabulary tools modify cognitive processing while eliciting emotional responses related to self-monitoring and the maintenance of learning confidence (Gordani, 2013). Studies of emotional affordance design in online learning environments further report that digital classrooms evoke immediate emotional reactions alongside delayed feedback-related emotions, leading learners to develop layered patterns of emotion regulation and motivational maintenance during task performance (Park and Lim, 2018). Across these lines of evidence, emotional functioning in foreign language learning appears to operate through more than one psychological channel. Some processes are tied to emotional activation during immediate participation and task execution, whereas others relate to sustained self-monitoring and confidence maintenance. This pattern provides a theoretical basis for distinguishing primary and secondary emotional vigilance and for examining how these parallel processes function within evaluative classroom environments.

### 2.4 From emotional processes to learning functioning: linking exposure, vigilance, and learning outcomes in an integrated model

Research on foreign language learning outcomes has consistently examined how ability differences, learning behaviors, and task performance interact in shaping achievement. Studies of language learning aptitude demonstrate that variation in phonological, lexical, and grammatical learning capacities influences oral attainment in foreign languages, leading to persistent individual differences in learning outcomes (Saito, 2017). Longitudinal classroom investigations further show that beginners' progress in decoding foreign language texts does not develop in a strictly linear manner but stabilizes into functional learning patterns through ongoing feedback and self-adjustment (Woore, 2009). Evidence from instructional tool research also indicates that electronic and paper-based annotations affect vocabulary learning and reading comprehension in different ways, as learners allocate attention and process information differently under distinct feedback conditions (Lee et al., 2015). Classroom-based vocabulary studies similarly report that levels of participation and contextual engagement influence whether learning behaviors translate into sustained learning gains (Peker et al., 2018). More recent assessment research suggests that the association between learning behaviors and achievement is not linear but shaped jointly by evaluative classroom structures and learners' orientations toward self-regulation (Xia and Wang, 2022). Related work in social and emotional learning further indicates that learners' interpretations of social norms, feedback signals, and others' expectations affect learning persistence and the quality of task completion (Yu and Jiang, 2017). Collectively, this body of evidence suggests that learning functioning depends not only on ability and strategy use but also on classroom contextual conditions and emotional processes that continuously shape learner engagement. On this basis, the present study investigates how contextual exposure in continuously evaluative classrooms relates to foreign language learning functioning through emotional vigilance and proposes a mechanism linking contextual exposure, emotional vigilance, and learning outcomes.

## 3 Methods

### 3.1 Research design

This study adopted a cross-sectional quantitative design to examine how perceived exposure to evaluative classroom conditions was associated with foreign language learning outcomes through the psychological process of emotional vigilance. Individual learners were treated as the primary unit of analysis. Within the proposed analytical framework, contextual exposure was specified as the antecedent condition, primary and secondary emotional vigilance were modeled as psychological mediating processes, and perceived learning outcome was treated as the dependent variable. In addition, age and gender-related background information were included as control variables, while a grouping variable was incorporated for subgroup comparison in order to assess whether the structural relationships remained stable across learner categories. Because all variables were measured at a single time point, the study was designed to identify structural associations and indirect psychological linkages rather than to establish temporal ordering or causal development over time. Accordingly, expressions such as “continuous evaluation,” “sustained exposure,” and “ongoing vigilance” in the present study refer to participants' perceived classroom conditions rather than to directly observed longitudinal processes.

### 3.2 Participants and setting

The study was conducted in China within a higher-education foreign language learning context. The final analytical sample consisted of 550 university students enrolled in foreign language courses or related language-learning programs. Participants ranged in age from 18 to 25 years, with a mean age of 21.01 years and a standard deviation of 1.27, indicating that the sample represented students of typical university age. The relatively narrow age range suggests a comparatively homogeneous student population in terms of educational stage, which helps reduce age-related heterogeneity in the interpretation of classroom experiences. In the subsequent analyses, age was entered as a control variable, and an additional grouping variable with an approximately balanced distribution was used for subgroup comparison. In light of the need for contextual transparency, the findings should be interpreted specifically within a Chinese university foreign language learning setting rather than as representative of the broader Asia-Pacific region.

### 3.3 Procedure

Data were collected through a self-administered questionnaire distributed to eligible university students in foreign language learning settings in China. Before participation, respondents were informed of the academic purpose of the study and were told that participation was voluntary, anonymous, and unrelated to course grading or institutional evaluation. They were also informed that they could discontinue participation at any time without penalty. All measures were completed during a single survey session. The questionnaire asked participants to report their perceptions of evaluative classroom exposure, their emotional vigilance during foreign language learning, and their perceived learning functioning under conditions characterized by feedback, visibility, monitoring, and comparison. Demographic information, including age and background variables, was collected at the end of the questionnaire. Prior to analysis, cases with substantial missing data or clearly invalid response patterns were removed, and the final valid sample retained for analysis comprised 550 responses.

### 3.4 Measures

All focal constructs were measured using multi-item Likert-type scales. Unless otherwise stated, all items were rated on a five-point response scale ranging from 1 (strongly disagree) to 5 (strongly agree), with higher scores indicating higher levels of the corresponding construct. To improve methodological transparency and reproducibility, the full wording of all items is provided in Supplementary Appendix A. Contextual exposure referred to students' perceived exposure to direct evaluative classroom conditions, including performance visibility, immediate feedback, comparison, and judgment during classroom participation, and this construct was assessed using six items with good internal consistency (Cronbach's α = 0.885). Primary emotional vigilance captured students' immediate alertness, attentional activation, and self-regulatory readiness in response to evaluative classroom cues, and it was measured with five items, also showing good reliability (Cronbach's α = 0.834). Learning outcome referred to students' self-reported learning functioning in the classroom context, including perceived task effectiveness, engagement stability, and learning performance under evaluative conditions, and it was measured using six items with good internal consistency (Cronbach's α = 0.862). Secondary exposure represented supplementary or relational forms of evaluative exposure embedded in classroom interaction, such as indirect comparison, social expectation, or diffuse monitoring cues, and it was measured with five items, yielding good reliability (Cronbach's α = 0.830). Secondary emotional vigilance reflected a broader and more ongoing form of self-monitoring, cautious participation, and relational adjustment across classroom situations, and it was measured using four items with acceptable internal consistency (Cronbach's α = 0.760). Although the reliability coefficient for secondary emotional vigilance was lower than those of the other focal variables, it remained within an acceptable range and may reflect the relatively broader and more heterogeneous conceptual scope of this construct. In addition to the focal variables, age was included as a continuous control variable, and binary-coded background variables were entered where appropriate in the regression and moderation analyses. The measurement items were developed for the present study on the basis of prior literature on evaluative classroom climate, emotional regulation, self-monitoring, and foreign language learning, and were adapted to fit the present research context.

### 3.5 Data analysis

Data analysis was conducted in Python 3.13.9 (Python Software Foundation, Wilmington, DE, USA) using the pandas, scipy, and statsmodels libraries. Descriptive statistics, including means, standard deviations, and observed score ranges, were calculated to summarize the distributional characteristics of the sample and the main constructs. Internal consistency reliability was then assessed through Cronbach's alpha coefficients for each measurement scale. To examine the basic associations among contextual exposure, emotional vigilance, and learning outcomes, Pearson correlation coefficients were estimated. The direct association between contextual exposure and learning outcome was subsequently tested using ordinary least squares regression while controlling for background variables. The indirect effect of contextual exposure on learning outcome through primary emotional vigilance was estimated through a bootstrap mediation analysis with 3,000 resamples, and 95% confidence intervals were used to evaluate the robustness of the indirect effect. To assess the explanatory contribution of the focal predictors, a baseline regression model including contextual exposure and secondary exposure was estimated, followed by a full model in which both primary and secondary emotional vigilance were entered simultaneously so that the additional explanatory value of emotional vigilance could be examined beyond exposure variables alone. The stability of these relationships across learner groups was further examined through moderation analyses that included the grouping variable together with the interaction terms between group membership and the two exposure variables. Statistical significance was evaluated using conventional thresholds (*p* < 0.05, *p* < 0.01, *p* < 0.001). In interpreting the regression results, particular attention was given to the possibility that secondary emotional vigilance might display a differentiated statistical role, given the contrast between its relatively weak zero-order correlations and its positive adjusted regression coefficient in the full model.

## 4 Results

### 4.1 Sample profile and descriptive properties of core constructs

Table 1 presents the sample characteristics and descriptive statistics of the study variables. Overall, the mean scores of the five core constructs range from 2.972 to 3.066, indicating that participants reported generally moderate levels of contextual exposure, emotional vigilance, and learning outcomes. This pattern suggests that evaluative classroom experiences were a relatively common feature of students' classroom participation rather than rare or highly extreme events. The standard deviations for the five main scale variables range from 1.043 to 1.110, indicating sufficient dispersion and suggesting that meaningful individual differences existed in how students perceived evaluative classroom conditions and responded to them psychologically. In addition, the observed score ranges show that all core variables span the full response scale from 1 to 5, indicating that the data captured both lower and higher levels of classroom exposure, vigilance, and learning-related perceptions and therefore provided adequate variability for subsequent analyses. Specifically, ContextExposure (M = 2.996, SD = 1.110), PrimaryMediator (M = 2.972, SD = 1.074), and Outcome (M = 3.025, SD = 1.055) are all located close to the midpoint of the scale, suggesting that the sampled students generally reported moderate evaluative exposure, moderate primary emotional vigilance, and moderate perceived learning functioning. SecondaryExposure (M = 3.015, SD = 1.087) and SecondaryMediator (M = 3.066, SD = 1.043) also fall near the center of the response range, indicating that secondary forms of exposure and vigilance were present at comparable levels. The control variables likewise show plausible and balanced distributions. Control_1 indicates that the participants were predominantly university students of typical college age (M = 21.009, SD = 1.270; range = 18–25), while Control_2 (M = 0.453, SD = 0.498) and Group (M = 0.469, SD = 0.499) display relatively even binary distributions, supporting their inclusion in subsequent regression and subgroup analyses. Taken together, the descriptive results suggest that the sample was characterized by moderate classroom-evaluative experiences and adequate variation across all main constructs, providing an appropriate empirical basis for the subsequent reliability, correlation, mediation, and moderation analyses.

**Table 1:** Sample characteristics and descriptive statistics of study variables.

Term	Mean	Std	Min	Max
Context exposure	2.996	1.110	1.0	5.0
Primary mediator	2.972	1.074	1.0	5.0
Outcome	3.025	1.055	1.0	5.0
Secondary exposure	3.015	1.087	1.0	5.0
Secondary mediator	3.066	1.043	1.0	5.0
Control_1	21.009	1.270	18.0	25.0
Control_2	0.453	0.498	0.0	1.0
Group	0.469	0.499	0.0	1.0

### 4.2 Internal consistency and scale reliability of measurement constructs

Table 2 reports the internal consistency reliability of the measurement scales. The Cronbach's alpha coefficients range from 0.760 to 0.885, indicating acceptable to good internal consistency across the five constructs. Contextual exposure shows good reliability (α = 0.885), suggesting that the six items measuring students' perceived exposure to direct evaluative classroom conditions form a coherent scale. Primary emotional vigilance (α = 0.834), learning outcome (α = 0.862), and secondary exposure (α = 0.830) also demonstrate good internal consistency, indicating that the corresponding items consistently capture the intended dimensions of emotional vigilance, perceived learning functioning, and supplementary forms of evaluative exposure. Secondary emotional vigilance yields a somewhat lower alpha coefficient (α = 0.760), although it remains within an acceptable range for research purposes. This relatively lower coefficient may reflect the broader and more heterogeneous conceptual scope of the construct, which appears to involve a less uniform and more differentiated form of self-monitoring than primary emotional vigilance. Overall, the reliability results suggest that the measurement scales used in the present study provide an adequate basis for the subsequent correlation, regression, mediation, and moderation analyses.

**Table 2:** Internal consistency reliability of measurement scales.

Construct	n_items	Cronbach_alpha
Context exposure	6	0.885
Primary mediator	5	0.834
Outcome	6	0.862
Secondary exposure	5	0.830
Secondary mediator	4	0.760

Cronbach's alpha coefficients ranged from 0.760 to 0.885, indicating acceptable to good internal consistency.

### 4.3 Zero-order associations among context exposure, emotional vigilance, and learning outcomes

Table 3 presents the Pearson correlation matrix among the core study variables. Overall, the results show a coherent pattern of associations among contextual exposure, emotional vigilance, and learning outcomes. Contextual exposure is positively correlated with primary emotional vigilance (*r* = 0.644, *p* < 0.001), indicating that higher levels of evaluative classroom exposure are associated with stronger immediate vigilance and attentional activation. Contextual exposure is also positively associated with learning outcome (*r* = 0.518, *p* < 0.001), while primary emotional vigilance is positively correlated with learning outcome (*r* = 0.681, *p* < 0.001), suggesting that students who report greater evaluative exposure and stronger primary vigilance also tend to report higher levels of perceived learning functioning. Secondary exposure shows significant positive correlations with contextual exposure (*r* = 0.369, *p* < 0.001), primary emotional vigilance (*r* = 0.475, *p* < 0.001), and learning outcome (*r* = 0.475, *p* < 0.001), indicating that different forms of evaluative exposure tend to co-occur and are jointly associated with stronger learning-related perceptions. In contrast, the pattern for secondary emotional vigilance is comparatively weaker and more differentiated. Secondary emotional vigilance is negatively correlated with contextual exposure (*r* = −0.201, *p* < 0.001), not significantly associated with primary emotional vigilance (*r* = −0.073, ns), not significantly associated with learning outcome (*r* = 0.041, ns), and not significantly related to secondary exposure (*r* = 0.014, ns). This configuration suggests that secondary emotional vigilance may represent a more distinct and less uniformly connected form of psychological regulation than primary vigilance. Whereas primary emotional vigilance appears closely tied to evaluative exposure and learning functioning, secondary emotional vigilance may operate more selectively and may reflect a broader pattern of cautious monitoring that is not directly aligned with the other dimensions of classroom exposure. Taken together, the correlation results indicate that evaluative classroom conditions are positively associated with heightened primary vigilance and stronger perceived learning outcomes, whereas secondary vigilance shows a more limited and differentiated pattern of association across the study variables.

**Table 3:** Pearson correlation matrix of core study variables.

Variable	1	2	3	4	5
Context exposure	1				
Primary mediator	0.644^***^	1			
Outcome	0.518^***^	0.681^***^	1		
Secondary exposure	0.369^***^	0.475^***^	0.475^***^	1	
Secondary mediator	−0.201^***^	−0.073 ns	0.041 ns	0.014 ns	1

*N* = 550.

^*^p < 0.05,

^**^p < 0.01,

^***^p < 0.001.

### 4.4 Direct association between context exposure and learning outcomes after covariate adjustment

Table 4 presents the regression results examining the direct association between contextual exposure and learning outcomes after controlling for demographic variables. The results show that contextual exposure remains a strong and statistically significant positive predictor of learning outcome (B = 0.574, SE = 0.032, *t* = 17.773, *p* < 0.001), indicating that students who reported higher levels of evaluative classroom exposure also tended to report higher levels of perceived learning functioning. In contrast, neither of the control variables reached statistical significance, as Control_1 showed a small negative but non-significant coefficient (B = −0.012, SE = 0.028, *t* = −0.416, ns) and Control_2 also remained non-significant (B = −0.125, SE = 0.072, *t* = −1.736, ns). This pattern suggests that the relationship between contextual exposure and learning outcome is not explained by basic demographic differences in the sample. Instead, the results point to the classroom evaluative environment itself as a key condition associated with students' perceived learning performance. At the same time, the positive coefficient for contextual exposure should not be interpreted as meaning that evaluative classroom conditions are uniformly easy or unproblematic. Rather, greater exposure appears to be associated with stronger adaptation to performance visibility, feedback, and external standards in classroom settings. Students in more evaluatively structured environments may therefore report higher learning functioning because they align their attention, participation, and learning efforts more closely with recognized classroom expectations. Taken together, the results provide support for a direct positive association between contextual exposure and perceived learning outcomes and establish an empirical basis for further examining whether emotional vigilance helps explain this relationship.

**Table 4:** Regression analysis of context exposure predicting learning outcomes.

Predictor	B	SE	*t*	Significance
Constant	1.607	0.601	2.675	^***^
Context exposure	0.574	0.032	17.773	^***^
Control_1	−0.012	0.028	−0.416	ns
Control_2	−0.125	0.072	−1.736	ns

*N* = 550. Dependent variable = outcome. ^*^p < 0.05, ^**^p < 0.01,

^***^p < 0.001.

### 4.5 Bootstrapped indirect effect of primary emotional vigilance linking context exposure to learning outcomes

**Figure 1:**
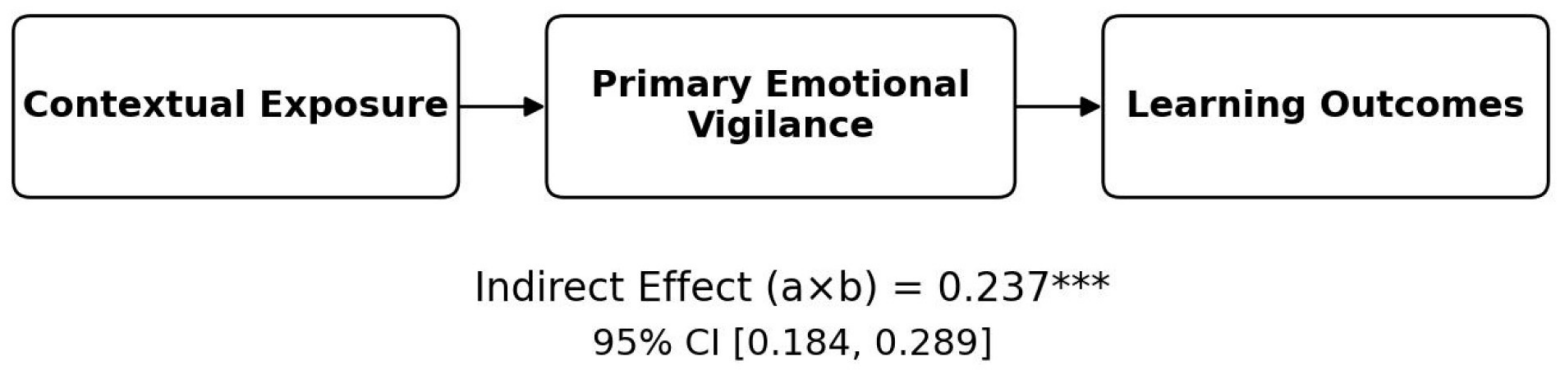
Mediation path diagram for primary emotional vigilance. Bootstrap confidence interval does not include zero. *N* = 550. ^***^p < 0.001.

Table 5 and Figure 1 present the bootstrapped indirect effect of contextual exposure on learning outcomes through primary emotional vigilance. The results show that this indirect pathway is statistically significant, with an estimated effect of 0.237 (SE = 0.027) and a 95% bootstrap confidence interval of [0.184, 0.289]. Because the confidence interval does not include zero, the mediation effect can be regarded as statistically reliable. This finding indicates that primary emotional vigilance functions as an important intervening mechanism linking evaluative classroom exposure to students' perceived learning outcomes. In other words, greater contextual exposure is associated not only with learning outcomes directly, but also indirectly through heightened primary emotional vigilance, including stronger attentional readiness and more immediate self-regulatory engagement in response to evaluative classroom conditions. The size of the indirect effect suggests that this mediating pathway accounts for a meaningful portion of the overall relationship between classroom exposure and perceived learning functioning. Overall, the results support the interpretation that the association between evaluative classroom conditions and learning outcomes is partly transmitted through students' primary emotional vigilance, highlighting the role of psychological regulation in shaping how classroom evaluation is linked to learning-related perceptions.

**Table 5:** Bootstrap estimates of the indirect effect via primary emotional vigilance.

Indirect path	Effect	SE	95% CI	Significance
Context exposure → Primary mediator → outcome	0.237	0.027	[0.184, 0.289]	^***^

^***^p < 0.001.

### 4.6 Baseline predictive model of learning outcomes: joint effects of primary and secondary exposure

Table 6 presents the baseline regression model predicting learning outcomes from contextual exposure and secondary exposure before the emotional vigilance variables are introduced. The results show that both contextual exposure and secondary exposure are significant positive predictors of learning outcomes, with coefficients of 0.463 (SE = 0.034, *t* = 13.675, *p* < 0.001) and 0.271 (SE = 0.035, *t* = 7.836, *p* < 0.001), respectively. This pattern indicates that both direct and secondary forms of evaluative classroom exposure are positively associated with students' perceived learning functioning. The coefficient for contextual exposure is larger than that for secondary exposure, suggesting that direct evaluative exposure has a relatively stronger association with learning outcomes at this stage of the analysis, although both contribute meaningfully to the model. The grouping variable does not reach statistical significance (B = −0.055, SE = 0.068, *t* = −0.808, ns), indicating that group membership does not independently explain variation in learning outcomes once the two exposure variables are taken into account. The model explains 43.2% of the variance in learning outcomes (*R*^2^ = 0.432, Adjusted *R*^2^ = 0.429), which suggests that exposure-related classroom conditions account for a substantial proportion of differences in students' reported learning functioning. Overall, the baseline model shows that evaluative classroom exposure is strongly associated with learning outcomes prior to the inclusion of emotional vigilance variables and therefore provides an important empirical foundation for examining whether vigilance further explains this relationship in the full model.

**Table 6:** Baseline regression model for learning outcomes.

Predictor	Coef.	SE	*t*	Significance
Constant	0.845	0.120	7.018	^***^
Context exposure	0.463	0.034	13.675	^***^
Secondary exposure	0.271	0.035	7.836	^***^
Group	−0.055	0.068	−0.808	ns
Observations (*N*)	550			
*R* ^2^	0.432			
Adjusted *R*^2^	0.429			

^***^p < 0.001.

### 4.7 Incremental contribution of emotional vigilance variables to perceived learning outcomes

**Figure 2:**
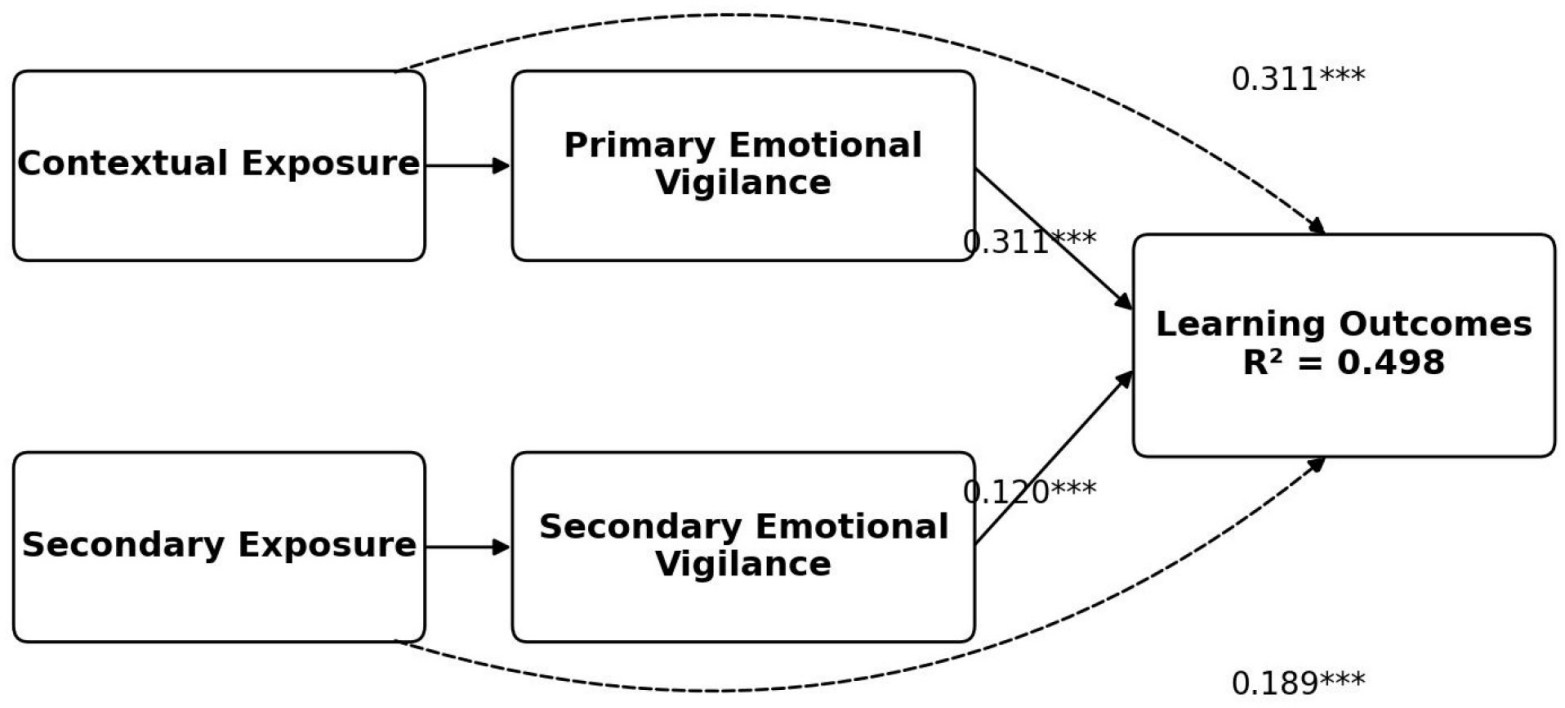
Integrated model of contextual exposure, emotional vigilance, and perceived learning outcomes. Dependent variable = outcome. *p < 0.05, **p < 0.01, ***p < 0.001.

Table 7 and Figure 2 present the full regression model after both forms of emotional vigilance are included alongside contextual exposure variables in predicting learning outcomes. The results show that the explanatory power of the model increases from *R*^2^ = 0.432 in the baseline model to *R*^2^ = 0.498 in the full model, indicating that the addition of the two emotional vigilance variables improves the model's ability to account for variation in learning outcomes. Within the full model, both primary emotional vigilance and secondary emotional vigilance are statistically significant positive predictors of learning outcomes. Primary emotional vigilance shows a coefficient of 0.311 (SE = 0.041, *t* = 7.597, *p* < 0.001), while secondary emotional vigilance shows a smaller but still significant coefficient of 0.120 (SE = 0.031, *t* = 3.853, *p* < 0.001). This pattern suggests that both forms of vigilance contribute positively to students' perceived learning functioning, although the effect of primary emotional vigilance is stronger. At the same time, contextual exposure and secondary exposure remain significant in the full model, with coefficients of 0.311 (SE = 0.039, *t* = 7.967, *p* < 0.001) and 0.189 (SE = 0.034, *t* = 5.558, *p* < 0.001), respectively. Compared with the baseline model, the coefficient for contextual exposure decreases from 0.463 to 0.311 and the coefficient for secondary exposure decreases from 0.271 to 0.189, indicating that part of the association between exposure variables and learning outcomes is accounted for by emotional vigilance. The constant term is not significant (B = 0.233, SE = 0.148, *t* = 1.572, ns), which does not affect the substantive interpretation of the main predictors. Overall, the full model shows that learning outcomes are jointly associated with evaluative classroom exposure and students' emotional vigilance, and the increase in explained variance supports the view that psychological regulatory processes provide additional explanatory value beyond contextual exposure alone. Notably, secondary emotional vigilance showed weak zero-order associations with several core variables but a significant positive adjusted coefficient in the full model. This pattern suggests a conditional association, possibly reflecting a suppression-like effect in which the adaptive component of secondary vigilance becomes more visible after overlapping variance with contextual exposure and primary emotional vigilance is taken into account.

**Table 7:** Regression model including emotional vigilance variables.

Predictor	Coef.	SE	*t*	Significance
Constant	0.233	0.148	1.572	ns
Context exposure	0.311	0.039	7.967	^***^
Secondary exposure	0.189	0.034	5.558	^***^
Primary mediator	0.311	0.041	7.597	^***^
Secondary mediator	0.120	0.031	3.853	^***^
*R* ^2^	0.498			
Adjusted *R*^2^	0.494			

^***^p < 0.001.

### 4.8 Stability of exposure–outcome links across groups: moderation tests for context exposure effects

Table 8 presents the moderation analysis examining whether the relationship between evaluative classroom exposure and learning outcomes differs across groups. The results show that both contextual exposure and secondary exposure remain significant positive predictors of learning outcomes, with coefficients of 0.459 (SE = 0.047, *t* = 9.696, *p* < 0.001) and 0.319 (SE = 0.048, *t* = 6.650, *p* < 0.001), respectively. In contrast, the main effect of group is not statistically significant (B = 0.220, SE = 0.231, *t* = 0.951, ns), and neither of the interaction terms reaches significance, as the interaction between contextual exposure and group shows a coefficient of 0.009 (SE = 0.068, *t* = 0.132, ns) and the interaction between secondary exposure and group shows a coefficient of −0.100 (SE = 0.069, *t* = −1.447, ns). These findings indicate that the positive association between evaluative classroom exposure and learning outcomes does not vary significantly across groups, suggesting that the predictive effects of classroom exposure are relatively stable within the sample. In other words, students in different groups appear to respond to evaluative classroom conditions in broadly similar ways, and the link between exposure and perceived learning functioning remains consistent rather than group-specific. The absence of significant interaction effects suggests that group membership does not meaningfully strengthen or weaken the association between classroom exposure and learning outcomes. Overall, the moderation results support the interpretation that evaluative classroom conditions exert a generally similar influence on learning-related perceptions across subgroups, reinforcing the robustness of the exposure–outcome relationship observed in the earlier models.

**Table 8:** Moderation analysis of group differences in context exposure effects.

Predictor	Coef.	SE	*t*	Significance
Context exposure	0.459	0.047	9.696	^***^
Secondary exposure	0.319	0.048	6.650	^***^
Group	0.220	0.231	0.951	ns
**Contextual exposure** × group	0.009	0.068	0.132	ns
**Secondary exposure** × group	−0.100	0.069	−1.447	ns
Observations (*N*)	550			
*R* ^2^	0.435			
Adjusted *R*^2^	0.429			

Bold values indicate statistically significant coefficients at *p* < 0.05.

^***^p < 0.001.

## 5 Discussion

### 5.1 Structural organization of learning in evaluative classrooms

Evaluative classrooms appear to provide more than informational feedback alone; they also constitute a relatively stable perceived contextual condition within which foreign language learning is organized. In the present sample, students' perceived levels of contextual exposure remained moderate, with mean values close to the midpoint of the scale, suggesting that evaluation was experienced less as an occasional instructional event than as a regular feature of everyday classroom interaction. Within such settings, perceived learning outcomes appear to be associated not only with learners' pre-existing characteristics, but also with how learners adjust their participation, attention, and performance strategies under conditions of visibility, comparison, and feedback. Consistent with this interpretation, contextual exposure showed a strong positive association with perceived learning outcomes (B = 0.574, *t* = 17.773, *p* < 0.001), and the baseline model indicated that exposure-related variables accounted for a substantial proportion of the variance in perceived learning outcomes (*R*^2^ = 0.432). These findings suggest that students who perceived stronger evaluative classroom conditions also tended to report stronger learning-related functioning within those same classroom environments. At the same time, this pattern should not be interpreted as indicating that evaluative conditions are uniformly facilitative or psychologically neutral. Rather, perceived continuously evaluative classrooms may be associated with learners' greater orientation toward correctness, publicly recognizable performance, and anticipated judgment, while also being linked to a higher likelihood of sustained self-monitoring. In this sense, students do not merely receive feedback as a supportive instructional resource; they may also internalize evaluative expectations as part of their ongoing learning regulation. This may help account for why higher perceived learning outcome ratings coexist with heightened psychological regulation in the same classroom context. From an SLA perspective, such a pattern is theoretically important because it suggests that evaluative classroom environments may be associated with performance organization and task-focused adjustment, while also carrying the potential to narrow exploratory expression, reduce willingness to take linguistic risks, and intensify the affective demands of participation. The present findings therefore invite a more differentiated understanding of classroom assessment in foreign language education: evaluation appears to be associated with stronger reported learning-related functioning, but this association may partly reflect learners' increased need to regulate themselves under persistent conditions of perceived judgment. This perspective extends existing discussions of assessment beyond its corrective and achievement-related functions and highlights its possible role in the psychological organization of classroom learning.

### 5.2 Differentiated psychological functions of primary and secondary emotional vigilance

Within continuously evaluative classroom environments, learners appear to display more than a single emotional response to judgment. Rather, the findings suggest that distinct forms of emotional vigilance operate simultaneously and perform different psychological functions in relation to learning outcomes. The mediation analysis indicates that primary emotional vigilance serves as a meaningful intervening mechanism linking contextual exposure to learning outcomes, with an indirect effect of 0.237 and a 95% confidence interval of [0.184, 0.289]. In the full model, primary emotional vigilance also shows a strong positive association with learning outcomes (B = 0.311, *t* = 7.597, *p* < 0.001). This pattern suggests that immediate attentional engagement, sensitivity to evaluative cues, and rapid self-adjustment are closely associated with how students function in evaluative classroom settings. In this sense, primary emotional vigilance appears to reflect a relatively direct form of regulatory alignment, through which learners organize participation around classroom standards, perceived correctness, and anticipated evaluation. Secondary emotional vigilance, by contrast, appears to follow a more differentiated functional logic. Although its predictive coefficient is smaller (B = 0.120, *t* = 3.853, *p* < 0.001), it remains statistically significant in the full model, even though its zero-order correlations with several core variables are weak or non-significant. This pattern suggests that secondary emotional vigilance may not represent a simple extension of primary vigilance, but rather a broader and more selective form of self-monitoring whose contribution becomes more visible after shared variance with contextual exposure and primary vigilance is taken into account. Substantively, it may reflect a more backgrounded orientation toward social awareness, cautious participation, and relational self-regulation in classroom interaction. Whereas primary vigilance appears more closely tied to immediate performance alignment, secondary vigilance may be more relevant to maintaining behavioral steadiness and emotional control in settings where learners remain aware of possible judgment even when it is not directly salient. Taken together, the presence of both forms of vigilance suggests that learning in evaluative foreign language classrooms involves not only attentional regulation in the moment, but also a more layered form of psychological management extending across participation contexts. This interpretation contributes to a more differentiated understanding of emotional processes in foreign language learning. Classroom emotional experience may not be adequately captured by single constructs such as anxiety or motivation alone; instead, multiple orientations of vigilance appear to operate together. At the same time, these findings should not be interpreted as suggesting that vigilance is uniformly beneficial. From an SLA perspective, heightened vigilance may support performance organization and responsiveness to evaluative demands, while also increasing self-consciousness and potentially narrowing opportunities for exploratory expression and linguistic risk-taking. The present results therefore point to a dual implication: evaluative classrooms may be associated with stronger reported learning functioning, but this association appears to be partly sustained through differentiated forms of psychological regulation that may also carry affective costs.

### 5.3 Cross-group stability of evaluative classroom effects and pedagogical implications

Across learner groups, the association between contextual exposure and learning outcomes appears relatively stable. Both contextual exposure and secondary exposure remain significant positive predictors of learning outcomes, whereas the group variable and the interaction terms do not reach statistical significance. This pattern suggests that the relationship between evaluative classroom exposure and perceived learning functioning does not vary substantially across the groups included in the present sample. In other words, students from different group backgrounds appear to respond to evaluative classroom conditions in broadly similar ways, and the predictive effects of classroom exposure remain comparatively consistent rather than group-specific. This finding is theoretically relevant because it suggests that evaluative classroom arrangements may operate as a shared organizational condition of participation, shaping students' learning-related perceptions through common structures of visibility, feedback, and comparison rather than through sharply differentiated subgroup mechanisms. At the same time, this relative stability should not be interpreted as evidence that learners are identical in experience or equally unaffected by classroom evaluation. Rather, the absence of significant moderation indicates that the exposure–outcome relationship remains broadly similar across groups, even though individual learners may still differ in capacity, comfort, and affective burden within the same classroom structure. From this perspective, evaluative classroom conditions may standardize certain forms of behavioral alignment by directing learners toward similar performance criteria and feedback expectations, while still allowing variation in how effortful or psychologically demanding that adaptation feels. This interpretation is especially important in foreign language learning, where stronger alignment with evaluative standards may support reported learning functioning but may also narrow the space for exploratory expression, meaning negotiation, and low-risk linguistic experimentation. The pedagogical implication is therefore not that evaluation should be minimized, but that assessment design should be calibrated more carefully. Feedback and evaluative guidance can support learning effectiveness, yet when evaluative cues become overly concentrated in everyday classroom interaction, participation may become too strongly oriented toward correctness, self-monitoring, and performance management. Effective foreign language assessment should therefore aim to preserve the benefits of feedback while also protecting opportunities for expressive flexibility, communicative risk-taking, and psychologically sustainable participation across different learner groups.

## 6 Limitations and conclusion

This study has several limitations that should be acknowledged. First, the research employed a cross-sectional questionnaire design, so the findings should be interpreted as evidence of patterned association rather than as direct proof of temporal development or causal change over time. In this sense, what is described in the present study as “continuous” or “ongoing” evaluation refers to learners' perceived experience of evaluative classroom conditions rather than to longitudinally observed change. Second, all focal variables were measured through self-report, which may introduce shared method variance and may not fully capture the interactional complexity of evaluative classroom processes as they unfold in real classroom settings. Third, the study was conducted within a Chinese university foreign language context, and the findings should therefore be interpreted with appropriate caution when considering other cultural, curricular, or institutional settings. Finally, although the measurement scales demonstrated acceptable to good internal consistency overall, the newly adapted constructs would benefit from further validation across additional samples and instructional contexts. These limitations do not invalidate the present findings, but they do suggest that the conclusions should be framed within the boundaries of the current design and sample.

Although these limitations should be kept in mind, the study still offers several meaningful contributions to the understanding of foreign language learning under evaluative classroom conditions. The findings suggest that evaluative classroom cues are experienced as a regular feature of participation rather than as isolated instructional events, and that stronger contextual exposure is positively associated with students' reported learning outcomes. More importantly, this relationship appears to operate not only directly, but also through differentiated forms of emotional vigilance. Primary emotional vigilance emerged as a significant mediating mechanism, while secondary emotional vigilance showed a smaller but still meaningful independent contribution in the full model, indicating that psychological regulation in evaluative classrooms is more layered than a single emotional construct can capture. In addition, the absence of significant moderation across learner groups suggests that the exposure–outcome relationship remains relatively stable within the present sample, supporting the view that evaluative classroom conditions function as a shared organizational feature of participation rather than a sharply group-specific process. At the same time, these results should not be interpreted as implying that vigilance is uniformly beneficial. From an SLA perspective, heightened vigilance may support attentional alignment and responsiveness to classroom demands, yet it may also intensify self-consciousness and reduce the space for exploratory expression and linguistic risk-taking. Taken together, the present findings suggest that classroom evaluation appears to function not only as a source of instructional feedback, but also as a perceived contextual condition associated with how learning is psychologically organized and regulated. This perspective has practical implications for foreign language assessment design, indicating that effective evaluation should aim to maintain feedback quality while also preserving opportunities for communicative flexibility, low-risk participation, and psychologically sustainable learning.

## Data Availability

The raw data supporting the conclusions of this article will be made available by the authors, without undue reservation.
