# Supplementary material for: Emotional vigilance under perceived continuous evaluation in foreign language classrooms: evidence from Chinese university students
Source: Front Psychol. 2026 May 12;17:1793299. doi: 10.3389/fpsyg.2026.1793299 (PMC13202636; doi:10.3389/fpsyg.2026.1793299)
Supplement: Supplementary file 1 [file Supplementary_file_1.docx]

Appendix A. Measurement Items Used in the Study

Response format. Unless otherwise stated, all items were rated on a five-point Likert scale ranging from 1 (strongly disagree) to 5 (strongly agree). Higher scores indicate higher levels of the corresponding construct.

A1. Contextual Exposure

This construct refers to students’ perceived exposure to direct evaluative classroom conditions, including performance visibility, immediate feedback, comparison, and judgment during classroom participation.

In my foreign language classroom, my classroom performance is usually clearly visible to others.

I often feel that my classroom responses are subject to immediate evaluation.

My teacher’s feedback makes me aware that my classroom performance is being continuously noticed.

During classroom participation, I feel that my responses are easily compared with those of other students.

I often feel that classroom interaction contains clear evaluative meaning.

In this foreign language course, evaluation is a common part of classroom activity.

A2. Primary Emotional Vigilance

This construct captures students’ immediate alertness, attentional concentration, and rapid self-regulatory adjustment in response to evaluative classroom cues.

When I realize that my classroom performance may be evaluated, I immediately become more alert.

When speaking in class, I quickly pay attention to whether what I say is appropriate.

As soon as I notice that my performance is being attended to, I immediately adjust my response.

In classroom situations where I may be evaluated, my attention becomes focused very quickly.

When I feel that I am in a situation of possible judgment, I immediately monitor my expression.

A3. Perceived Learning Outcomes

This construct reflects students’ subjective perceptions of their learning-related functioning, including task completion, stability of participation, and perceived effectiveness in classroom learning.

I am able to participate in foreign language classroom activities in a stable way.

In this foreign language course, I am usually able to complete classroom learning tasks effectively.

I feel that my engagement in classroom learning is generally effective.

I believe that I maintain a good level of concentration in this foreign language class.

I feel that I have made noticeable learning progress in this foreign language course.

Overall, I think my learning performance in this foreign language class is satisfactory.

A4. Secondary Exposure

This construct refers to indirect, relational, or diffuse forms of evaluative exposure embedded in classroom interaction, such as implicit comparison, social expectation, and subtle monitoring cues.

Even when the teacher is not evaluating me directly, I can still sense an evaluative atmosphere in the classroom.

Even when it is not stated explicitly, I can sense comparison among students in class.

Other people’s reactions in class make me aware that my performance may be judged.

I often feel that evaluation is present in classroom interaction in indirect ways.

Social expectations in the classroom make me more aware of the way I present myself as a learner.

A5. Secondary Emotional Vigilance

This construct reflects a more sustained and backgrounded form of self-monitoring and cautious adjustment, involving relational awareness and impression management during classroom participation.

In foreign language class, even when no one is evaluating me directly, I still pay attention to the impression I may leave on others.

I tend to monitor the way I perform throughout classroom participation.

In order to avoid negative evaluation, I consciously adjust my classroom behavior.

During classroom participation, I usually maintain a background awareness of the possibility of being judged.
